# *HLA-G* 14-bp variant is associated with exercise-induced oxygen desaturation in the post-COVID-19 condition

**DOI:** 10.1183/23120541.01038-2023

**Published:** 2024-07-15

**Authors:** Ingrid Fricke-Galindo, Paola Montoya-Angulo, Ivette Buendía-Roldán, Gloria Pérez-Rubio, Leslie Chávez-Galán, Ramcés Falfán-Valencia

**Affiliations:** 1 HLA Laboratory, Instituto Nacional de Enfermedades Respiratorias Ismael Cosio Villegas, Mexico City, Mexico; 2 Licenciatura en Químico Farmacéutico Biotecnólogo, Universidad del Valle de México, Mexico City, Mexico; 3 Translational Research Laboratory on Aging and Pulmonary Fibrosis, Instituto Nacional de Enfermedades Respiratorias Ismael Cosío Villegas, Mexico City, Mexico; 4 Laboratory of Integrative Immunology, Instituto Nacional de Enfermedades Respiratorias Ismael Cosío Villegas, Mexico City, Mexico

## Abstract

*HLA-G* (major histocompatibility complex, class I, G; HUGO Gene Nomenclature Committee identifier: 4964) belongs to the nonclassical *HLA-I* genes initially related to maternal–fetal tolerance; however, anti-inflammatory functions and modulation of the immune response have also been described [1]. *HLA-G* variants and plasma levels of the molecule have been investigated in autoimmune and infectious diseases [2]. In COVID-19, some patients experienced immune dysregulation, leading to excessive production of inflammation molecules, triggering the severe and critical forms of the disease [3].

## Shareable abstract

**The *HLA-G* 14-bp indel (rs371194629) variant is associated with IMV requirement in COVID-19 and with exercise-induced desaturation in the post-COVID-19 condition** https://bit.ly/3THdx1D

*To the Editor:*

*HLA-G* (major histocompatibility complex, class I, G; HUGO Gene Nomenclature Committee identifier: 4964) belongs to the nonclassical *HLA-I* genes initially related to maternal–fetal tolerance; however, anti-inflammatory functions and modulation of the immune response have also been described [1]. *HLA-G* variants and plasma levels of the molecule have been investigated in autoimmune and infectious diseases [2]. In COVID-19, some patients experienced immune dysregulation, leading to excessive production of inflammation molecules, triggering the severe and critical forms of the disease [3]. Higher plasma levels of the soluble form of HLA-G (sHLA-G) have been found in patients with COVID-19 compared to uninfected control subjects [4, 5]; the levels increased in patients with severe disease but decreased in critical patients [6]. The 14-bp insertion/deletion (indel) polymorphism, found in the 3′-untranslated region of the *HLA-G* gene, has been linked to the mRNA stability and the amounts of the sHLA-G plasma levels, with the lowest levels observed in those homozygous for the insertion (Ins) allele (Ins/Ins) [7]. The indel variant has been controversially associated with COVID-19 susceptibility [5] and severity [8]. However, its association with the disease's mortality and post-COVID-19 condition has not been reported. Herein, we assessed the association of the 14-bp indel *HLA-G* variant with COVID-19 susceptibility, severity and mortality, and its role in the post-COVID-19 in subjects recovering from a severe form of the disease.

Thus, three main groups were evaluated: 1) 621 patients with severe COVID-19 (determined by the presence of dyspnoea, respiratory rate ≥30 breaths per minute, blood oxygen saturation (*S*_pO__2_) ≤90% and/or arterial oxygen tension/inhaled oxygen fraction ratio (*P*_aO__2_/*F*_IO__2_) ≤300 mmHg at hospital admission) hospitalised in the Instituto Nacional de Enfermedades Respiratorias Ismael Cosio Villegas, Mexico City, Mexico; 2) 520 non-COVID-19 subjects (healthy volunteers recruited after the COVID-19 pandemic); and 3) 101 patients that were followed 12 months after discharge for COVID-19. COVID-19 diagnosis was established with a positive result on SARS-CoV-2 reverse transcriptase PCR. The severity of COVID-19 was evaluated through the invasive mechanical ventilation (IMV) requirement, while mortality was assessed as in-hospital mortality. The genetic association studies and the logistic regression models were performed in PLINK version 1.07 [9], while comparing variables between groups was evaluated in RStudio [10]. Significant p-values were corrected for multiple comparisons through the Bonferroni method. Normal distribution was assessed through the Kolmogorov–Smirnov test. Continuous data are presented as medians and interquartile ranges.

The clinical and demographic data of the studied groups are presented in table 1. Significant differences were observed for sex, body mass index (BMI) and smokers between the COVID-19 and non-COVID-19 groups; age and steroid administration between IMV groups; and age, sex and BMI were different between the survivors and nonsurvivors.

**TABLE 1:** Clinical and demographic data of the studied groups and genetic association studies of the *HLA-G* 14-bp insertion (Ins)/deletion (Del) variant (rs371194629) with COVID-19 susceptibility, severity and mortality, and in patients with post-COVID-19 condition

Variable	COVID-19	Non-COVID-19	IMV	Non-IMV	Nonsurvivors	Survivors	Desaturators^#^	Nondesaturators
**Subjects, n**	621	520	417	200	160	446	46	51
**Age, years**	55 (46–66)	53 (47–62)	**56** **(47–67)**	**54** **(44–64)**	**63** **(53–72)**	**54** **(44–63)**	58 (53–65)	54 (43–61)
**Males**	**400** **(64.4)**	**239** **(46.0)**	276 (66.2)	121 (60.5)	**117** **(73.1)**	**275** **(61.6)**	22 (47.8)	15 (29.4)
**BMI, kg·m^−2^**	**28.7** **(25.9–32.9)**	**27.2** **(24.5–30.2)**	28.8 (26–32.9)	28.6 (25.7–32.8)	**28.9** **(25.0–31.2)**	**29.1** **(26.2–33.1)**	**29.0** **(26.7–32.0)**	**27.3** **(25–29.9)**
**Smoking**	**201** **(32.4)**	**263** **(50.6)**	135 (32.4)	65 (32.5)	55 (34.3)	140 (31.5)	12 (26.1)	16 (31.4)
**Steroid administration**	496 (79.9)	NA	**356** **(85.3)**	**137** **(68.5)**	136 (85.0)	348 (78.0)	NA	NA
**CRD**	41 (6.6)	NA	23 (5.5)	18 (9.0)	11 (6.9)	29 (6.5)	8 (17.4)	8 (15.7)
**SAH**	223 (35.9)	NA	150 (36.0)	73 (36.5)	62 (38.7)	155 (34.8)	13 (28.3)	11 (21.6)
***HLA-G*^¶^** **rs371194629**								
Del	724 (0.582)	572 (0.550)	497 (0.596)	222 (0.555)	192 (0.600)	514 (0.576)	58 (0.630)	52 (0.510)
Ins	518 (0.417)	466 (0.448)	337 (0.404)	178 (0.445)	128 (0.400)	378 (0.424)	34 (0.369)	50 (0.490)
Del/Del	214 (0.345)	157 (0.302)	155 (0.372)	58 (0.290)	61 (0.381)	148 (0.332)	**20** **(0.435)**	**10** **(0.196)**
Del/Ins	296 (0.477)	258 (0.496)	187 (0.448)	106 (0.530)	70 (0.437)	218 (0.489)	**18** **(0.391)^+^**	**32** **(0.627)**
Ins/Ins	111 (0.179)	104 (0.200)	75 (0.180)	36 (0.180)	29 (0.181)	80 (0.179)	**8** **(0.174)^+^**	**9** **(0.176)**
Ins/Ins	111 (0.179)	104 (0.200)	75 (0.180)	36 (0.180)	29 (0.181)	80 (0.179)	8 (0.174)	9 (0.176)
Del/Del+Del/Ins	510 (0.821)	415 (0.798)	342 (0.820)	164 (0.820)	131 (0.819)	366 (0.821)	38 (0.826)	42 (0.823)
Del/Ins+Ins/Ins	407 (0.655)	362 (0.696)	**262** **(0.628)**	**142** **(0.710)**	99 (0.619)	298 (0.668)	**26** **(0.565)**	**41** **(0.804)**
Del/Del	214 (0.345)	157 (0.302)	**155** **(0.372)^§^**	**58** **(0.290)**	61 (0.381)	148 (0.332)	**20** **(0.435)^ƒ^**	**10** **(0.196)**

Data are presented as median (interquartile range), n (%) or n (allele or genotype frequency) unless otherwise stated. Bold indicates statistical significance. The study of the alleles and genotype frequencies, as well as the genetic models, were performed in PLINK v1.07. For the genetic models, Del was considered the risk allele. The Bonferroni method was employed for multiple comparison correction (p_c_). p>0.05 unless otherwise stated. The IMV, non-IMV, nonsurvivor and survivor groups included COVID-19 patients, while the desaturator and nondesaturator groups included subjects suffering from the post-COVID-19 condition. IMV: invasive mechanical ventilation; BMI: body mass index; CRD: chronic respiratory disease; NA: not applicable. ^#^: subjects were classified as desaturators when the oxygen saturation decreased >4% after the 6-min walking test performed 6 months after discharge; SAH: systemic arterial hypertension.^¶^: *HLA-G* rs371194629 genotyping was determined by end-point PCR, employing the oligonucleotides reported by Ad'hiah and Al-Bayatee [5]; the amplification was visualised on 2.5% agarose gel electrophoresis (Del: 210-bp fragment; Ins: 224-bp fragment).^+^: p=0.030, p_c_=0.120; Del/Ins OR 0.28 (95% CI 0.11–0.73), Ins/Ins OR 0.44 (95% CI 0.13–1.50). ^§^: p=0.046, p_c_=0.184; OR 1.45 (95% CI 1.01–2.08). ^ƒ^: p=0.011, p_c_=0.044; OR 3.15 (95% CI 1.28–7.79).

101 patients who presented severe COVID-19 were followed for a year (with follow-up every 3–4 months) because they showed a pulmonary dysfunction determined by a decrease in their forced vital capacity or desaturation in the 6-min walking test (6MWT) and/or the presence of interstitial thickening on computed tomography, which has been previously reported in other cohorts [11, 12]. The tests performed in each follow-up were spirometry, diffusing capacity of the lung for carbon monoxide and 6MWT. The clinical and demographical characteristics of this group are worth mentioning: 67 were males (66.3%), with a median (interquartile range) age 56 (49–64) years; 24 (23.8%) were smokers; and 18.8% presented at least one comorbidity (19 with type 2 diabetes, 15 with systemic arterial hypertension and 11 with chronic respiratory diseases). During their hospitalisation due to COVID-19, the symptom frequencies were similar to those previously reported in patients with COVID-19 [13]: *P*_aO__2_/*F*_IO__2_ at hospital admission was 151 (103–187) mmHg; all of them required IMV, for 17 (9–24) days; and hospital stay was 27 (17–35) days.

The allele and genotype frequencies of the 14-bp indel *HLA-G* variant in each studied group are shown in table 1. The allele and genotype frequencies were similar for the susceptibility, severity, and mortality of COVID-19 assessments. In each association study, we performed the Bonferroni method for multiple-comparison correction and logistic regression models for covariable adjustment (the clinical and demographical variables were significantly different between the groups, which can be observed in table 1), but no significant values were found. In the post-COVID-19 group, a difference in the genotype frequencies was observed when compared between desaturators and nondesaturators on the 6MWT performed 6 months after discharge. The heterozygous deletion (Del)/Ins genotype was found to be a low-risk genotype for desaturation after 6MWT (table 1), even after adjusting for BMI in the binary logistic regression (p=0.036); however, it was not significant after Bonferroni correction.

We also performed the analyses using genetic models (table 1) and we observed that, under the recessive model, the genotype Del/Del was more frequent among patients requiring IMV than those not requiring IMV, but the corrected p-value was no longer significant. We found that survivor patients requiring IMV during hospitalisation and carrying Del/Del genotype had a slightly higher number of IMV days (16 (11–20) *versus* 13 (9–17) days, p=0.049) and hospitalisation stay (27 (18–41) *versus* 21 (16–33) days, p=0.052) than patients carrying Del/Ins or Ins/Ins. Nevertheless, we found no differences in *P*_aO__2_/*F*_IO__2_ among the *HLA-G* genotypes.

In the post-COVID-19 condition, the Del/Del genotype of the recessive model was firstly associated with a three-fold desaturation risk after 6MWT performed 6 months after discharge (table 1). In contrast, the Del/Ins+Ins/Ins genotypes were associated with a low risk of exercise-induced desaturation in the regression model adjusted for BMI (OR 0.30, 95% CI 0.11–0.75; p=0.012). Even *S*_pO__2_ after the 6MWT were lower in Del/Del carriers than Del/Ins or Ins/Ins carriers, and this was observed for the first follow-up (87.5% (86–91%) *versus* 91% (88–93%), p=0.033), the second follow-up (87% (83–90%) *versus* 89% (87–91%), p=0.046) and the third follow-up (88% (87–89%) *versus* 90% (88–91%), p=0.004), but not for the fourth follow-up (88% (85.5–89%) *versus* 90% (85.5–90.5%), p=0.175). The remaining values of the pulmonary tests were not different between the *HLA-G* genotypes.

*HLA-G* is notably expressed in the lung and is involved in immune functions. Its relevance in COVID-19 has been previously reported [4–6], as we observed the association of the *HLA-G* 14-bp indel variant with the requirement of IMV. The marginal association with the length of IMV days and hospitalisation stay suggests a relevant role of *HLA-G* in pulmonary function, as it was also observed in the desaturation after 6MWT in post-COVID-19 patients. Further investigations are warranted to elucidate the mechanisms involving the *HLA-G* in the recovery of pulmonary function. However, the regulation of *HLA-G* expression mediated by oxygen and hypoxia-inducible factor 1 has been described [14, 15].

This study presents some limitations. It lacks a group of patients with asymptomatic to moderate COVID-19; thus, the susceptibility study is more related to the risk of a severe form of COVID-19. Moreover, using Bonferroni correction limits the association results by its conservative nature; although it can reduce the risk of false positives, it also increases the risk of false negatives. These findings require validation through replication in a similar cohort or mechanistic validation (*i.e.* experimental modelling).

This is the first evaluation of the *HLA-G* 14-bp indel (rs371194629) in the follow-up of patients with post-COVID-19 condition.

## Data Availability

ClinVar submission SCV004101717.
